# Predictive effect of sustainable dietary and literacy patterns on metabolic syndrome and diabetes risk in Turkish adults: Mediterranean diet, sustainable healthy eating behaviors, and sustainable food literacy perspective

**DOI:** 10.3389/fmed.2025.1693180

**Published:** 2025-10-03

**Authors:** Serap İncedal Irgat, Hande Bakırhan, Yunus Emre Bakırhan

**Affiliations:** ^1^Department of Nutrition and Dietetics, Faculty of Health Sciences, Karamanoğlu Mehmetbey University, Karaman, Türkiye; ^2^Department of Nutrition and Dietetics, Faculty of Health Sciences, Kahramanmaraş Istiklal University, Kahramanmaraş, Türkiye; ^3^Department of Nutrition and Dietetics, Faculty of Health Sciences, Izmir Katip Çelebi University, Izmir, Türkiye

**Keywords:** metabolic syndrome, diabetes, sustainable healthy eating behaviors, Mediterranean diet, sustainable food literacy

## Abstract

**Background:**

Food literacy and sustainable patterns may be associated with metabolic disease risk. Therefore, it is important to determine the potential impact of sustainable dietary concepts, as well as the development of food literacy, on the risk of metabolic syndrome (MetS) and diabetes. The aim of this study was to investigate the associations of sustainable dietary concepts and sustainable food literacy with the risk of MetS and diabetes in adults.

**Methods:**

This study included a total of 6,364 healthy Turkish adults. To determine the extent to which sustainable dietary concepts affect MetS and diabetes in participants, the status of participants was assessed with the following scales: the Sustainable and Healthy Eating Behaviors Scale (SHEBS), the Sustainable Food Literacy Scale (SFLS), the Mediterranean Diet Adherence Screener (MEDAS), the Metabolic Syndrome Index (MSI), the Metabolic Syndrome Research Form (MSAF), and the Finnish Diabetes Risk Score (FINDRISC).

**Results:**

Most participants were found to have a low risk of diabetes (62.6%), 49.8% had a moderate risk of MetS (based on the MSAF), and 21.3% had a high risk of MetS (based on the MSI). Participants with a high level of MEDAS had a lower MSI score than did those with a moderate or low level (*p* < 0.001), whereas those with a low level of MEDAS had a significantly higher MSAF score than did those with a moderate or high level (*p* < 0.001). As participants’ MSI and FINDRISC levels decreased, their SHEBS and SFLS scores significantly increased (*p* < 0.001). As MSAF levels increased, participants’ MEDAS scores significantly decreased (*p* < 0.001). SFLS and MEDAS had a negative and significant effect on the MSAF (*β* = −0.03, β = −0.04; *p* < 0.05, respectively), whereas SHEBS had a stronger and negatively significant effect (β = −0.08; *p* < 0.001). The MEDAS (*β* = −0.03; *p* = 0.007), SHEBS (β = −0.08; *p* < 0.001), and especially the SFLS (β = −0.13; *p* < 0.001) were found to be negative and significant predictors of MetS risk (for MSI), whereas the SFLS was a negatively significant predictor of diabetes risk (β = −0.11; *p* < 0.001).

**Conclusion:**

The effects of sustainable healthy eating behaviors, sustainable food literacy and Mediterranean diet on preventing the risk of MetS are significant, and the most important negative predictor of diabetes risk is SFLS.

## 1 Introduction

Metabolic syndrome (MetS) is a complex process that involves the intersection of risk factors such as abdominal obesity, glucose intolerance, hypertension, and dyslipidemia and particularly defines the risks associated with cardiovascular disease (CVD) and type 2 diabetes (T2DM) (1). While the prevalence of MetS worldwide varies between 7.9 and 43% in men and between 7 and 56% in women (2), it is known to be between 20 and 40% in Western societies (3). In Türkiye, the incidence of MetS is 1 in every 4 men and 1 in every 3 women, and its prevalence is 43.3% in the general population, 50.4% in women and 35.4% in men (4). The latest Diabetes Atlas of the International Diabetes Federation (IDF) reported that 11.1% of the adult population (1 in 9 and 589 million) are living with diabetes and that this number is predicted to increase to 853 million by 2050 (5). MetS and T2DM are at the forefront of global public health problems. An unhealthy diet and low physical activity play significant roles in the etiopathogenesis of MetS (6). Although improving nutritional habits and adopting a healthy lifestyle are considered fundamental strategies for the treatment and management of MetS, there is no consensus yet on the most effective dietary pattern in this regard (7). Despite this, the Mediterranean diet (MD) and Dietary Approaches to Stop Hypertension (DASH) are frequently recommended diets for patients with MetS, CVD and T2DM (6).

It has been suggested that there is a relationship between nutritional literacy and sustainable dietary behaviors and the risk of developing CVD and T2DM (8). Sustainable food consumption patterns and dietary behaviors are believed to have beneficial effects on human health, in addition to their positive impact on the environment. Therefore, by adopting a healthier diet and promoting the consumption of culturally appropriate, nutritious, local, and seasonal foods, we can be one step closer to achieving the goals of reversing the global health crisis (9, 10). The positive impact of MD on metabolic health, owing to its anti-inflammatory and antioxidant properties, bioactive components, unsaturated fats, polyphenols and fibers, together with lifestyle changes, stand out as a sustainable method to address the global burden of MetS and T2DM (11–14). Similarly, the role of MD in preventing T2DM and MetS through beneficial effects on cardiometabolic disease risk factors has been highlighted (14). Although studies examining the associations between dietary characteristics and the risk of MetS and T2DM have shown that healthier diets are associated with a lower risk of MetS and T2DM (11–13, 15), no study has holistically examined sustainable healthy eating patterns (sustainable healthy eating behaviors, MD characteristics) and sustainable food literacy concepts. To our knowledge, studies investigating the impact of these concepts on the risk of MetS and T2DM are limited. There are no studies examining this topic holistically. In particular, the impacts of MD, sustainable healthy eating behaviors, and sustainable food literacy on the risk of these diseases and their components in the Turkish population are not yet fully understood. Because these factors are more easily modifiable and ameliorable, determining their potential association with the risk of MetS and T2DM may be an important strategy to reduce the risk of morbidity and mortality from MetS and T2DM. We focus on the possibility that a sustainable healthy eating behavior approach and sustainable food literacy, along with an MD, which is a sustainable dietary pattern (13), may mitigate MetS and T2DM. It was hypothesized in this study that following an MD, which is defined as a sustainable diet (13), having sustainable healthy eating behaviors, and having a good level of sustainable food literacy may play an important role in metabolic regulation and metabolic-based risks. In this context, the aim of this study was to investigate the relationships between the risk of MetS and T2DM in Turkish adults and sustainable healthy eating patterns (sustainable healthy eating behaviors, MD characteristics) and sustainable food literacy.

## 2 Materials and methods

### 2.1 Study design and sample selection

The sample size of the study was calculated as 6,364 individuals in total, based on 99% power, an effect size of 0.085 and *α* = 0.05, via the biostatistical power analysis program G-Power. This cross-sectional study was conducted in Türkiye between March and June 2025 via a voluntary sampling method. The exclusion criteria included individuals under the age of 18 years or over the age of 65 years; those with a history of chronic CVDs such as stroke, myocardial infarction, coronary artery bypass, percutaneous coronary intervention, coronary angiography, heart failure, or peripheral artery disease; those with cancer, liver, or kidney disease; those with diagnosed metabolic syndrome and type 2 diabetes; those in a special diet program; pregnant or breastfeeding women; and those with a history of alcohol or drug addiction. A total of 6,364 healthy adults aged 18–65 years who did not meet any of the exclusion criteria and agreed to participate were included in the study. The participants were included in the study on a voluntary basis through open calls made through online platforms (Facebook, Instagram, WhatsApp, etc.). To enhance the representativeness of the sample, participants were invited from individuals of diverse socioeconomic backgrounds who live in various cities and districts in Türkiye. The participants who volunteered were informed about the study’s aim and scope via an online written statement, and their consent was obtained. Data collection tools were uploaded to the Google survey system, survey links were shared on social media platforms to provide wide access online, and the participants answered the online questionnaire through Google Forms.

### 2.2 Data collection

Data were obtained on the basis of participants’ self-reports. The online survey included questions on participants’ sociodemographic characteristics (age, sex, marital and educational status, profession, and anthropometric measurements such as height and weight). Anthropometric measurements (body weight, height, and waist circumference) were obtained through self-reporting. Participants were provided with written instructions on how to perform the measurements. Body mass index was calculated by the researchers. The online survey also includes six main scales: the Sustainable and Healthy Eating Behaviors Scale (SHEBS), the Sustainable Food Literacy Scale (SFLS), the Mediterranean Diet Adherence Screener (MEDAS), the Metabolic Syndrome Index (MSI), the Metabolic Syndrome Research Form (MSAF), and the Finnish Diabetes Risk Score (FINDRISC).

### 2.3 Assessment of diabetes risk

To assess the risk of T2DM, the FINDRISC, developed by Lindström and Tuomilehto and validated in Turkish and recommended for use in diabetes screening in adults by the Turkish Society of Endocrinology and Metabolism, was used (16, 17). The FINDRISC was chosen because it is easy to apply, valid in many European populations, including Turkey, and it can predict the 10-year risk of T2DM in adults on the basis of clinical and lifestyle factors. It consists of a total of eight questions and includes basic information such as age, BMI, physical activity status, medication use, fruit and vegetable consumption and family history. The possible scores on the scale range from 0 to 26. A total score of less than 7 indicates a very low 10-year risk of Type 2 DM, a score of 7–11 indicates low risk, a score of 12–14 indicates moderate risk, a score of 15–20 indicates high risk, and a score of more than 20 indicates very high risk (16, 17).

### 2.4 Assessment of metabolic syndrome risk

MetS risk was assessed via two different scales. The MSI, a tool developed by Akeren and Apaydın for the Turkish population and validated and reliable, was used to identify individuals at metabolic risk early. A total of 21 risk factors are evaluated, and the total score ranges from 0 to 100. It includes information such as age, BMI, waist circumference, presence of chronic disease, family history, presence of various metabolic symptoms such as headache and chest pain, sleep quality, physical activity status, alcohol and cigarette use, stress level, bread preference, legume consumption, fluid intake, and number of meals. Higher scores indicate a greater risk of MetS (18).

Another assessment tool used to assess MetS risk is the MSAF. The MSAF is a valid and reliable scale containing 14 questions developed by Erdoğmuş; it is preferred for determining the risk of MetS in Türkiye and has been used in various studies (19–21). It includes questions examining eating habits, body weight, physical activity, blood pressure, and abdominal and waist fat status. The total score ranges from 0 to 14, and as the score increases, the risk of MetS also increases. A score between 0 and 4 indicates low risk, 5 to 8 indicates moderate risk, and 9 to 14 indicates high risk of MetS (19, 20).

### 2.5 Assessment of Mediterranean diet characteristics

MD characteristics were assessed via the 14-item MEDAS developed by Martínez-González, which has been validated and reliably confirmed in Türkiye (22, 23). The MEDAS consists of 12 questions about food consumption frequency and 2 questions about food consumption habits, and a total score is obtained by taking 1 or 0 points from each question. A MEDAS total score of ≤5 was considered to indicate low MD features, a score between 6 and 9 indicated moderate MD features, and a score of ≥10 indicated good MD features (10, 22, 24).

### 2.6 Assessment of sustainable healthy eating behaviors

The Turkish version of the SHEBS, developed by Żakowska-Biemans et al. (25), whose adaptation, validity, and reliability studies were conducted by Köksal et al. (26), was used to assess sustainable and healthy eating behaviors. The SHEBS includes 32 questions and 7 subcomponents (quality labels, seasonal food & avoiding food waste, animal welfare, meat reduction, healthy & balanced nutrition, local food and low fat), and each component is answered on a 7-point Likert scale. While the score of each factor is calculated by taking the average of the scores of the relevant questions (between 1 and 7), the total scale score is calculated by taking the average of the scores of all factors (between 1 and 7). A higher total score indicates more sustainable and healthy eating behaviors (26).

### 2.7 Assessment of sustainable food literacy

The SFLS, developed by Teng and Chih (27) and whose Turkish validity and reliability study was conducted by Kubilay and Yüksel (28), was used to measure the ability to understand sustainable diets. The scale consists of 5 basic components, namely, sustainable food knowledge-I, sustainable food knowledge-II, food and culinary skills, attitudes, action intentions and action strategies, and all the items are rated on a seven-point Likert scale ranging from “strongly disagree” (1) to “strongly agree” (7). The possible scores from the scale range from 26 to 182, with increasing scores indicating a higher level of sustainable food literacy (28).

### 2.8 Internal consistency assessment

To increase the credibility of the results, internal consistency for each scale was assessed using the Cronbach’s *α* coefficient. In this study, the internal consistency coefficients for the scales were found to be 0.66 for the MEDAS, 0.93 for the SHEBS, 0.94 for the SFLS, 0.65 for the MSI, and 0.67 for the MSAF.

### 2.9 Data analysis

The data were analyzed via IBM SPSS Statistics 26.0 and AMOS 24.0. Descriptive statistics included means, standard deviations, frequencies, and percentage distributions. The suitability of the scales used for normal distribution was evaluated by examining the skewness and kurtosis coefficients; it was determined that all scales and subfactors met the normal distribution assumption. For categorical variables, the chi-square test was used, variables with two groups were analyzed with the independent samples t test, and variables with three or more groups were analyzed with one-way analysis of variance (one-way ANOVA). In cases where a significant difference was found as a result of the ANOVA test, *post hoc* comparison tests were applied to determine the level of difference between the groups. Relationships between scales and their subdimensions were evaluated via Pearson correlation analysis. The possible effects on the risk of T2DM and MetS were examined via path analysis via structural equation modeling. In path analysis, the explained variance ratios (R^2^) of the dependent variables were evaluated together with the standardized (*β*) and unstandardized (B) coefficients. For all the statistical analyses, the significance level was set at *p* < 0.05.

## 3 Results

The mean age of the participants was 27.0 ± 10.24 years, and the majority were female (60.4%), high school graduates (62.7%), single (73.7%), middle-income (55.7%), or students (54.0%) (Table 1). When the diet characteristics were examined, 62.5% (*n* = 3,980) exhibited moderate MD characteristics, 24.2% (*n* = 1,537) presented poor MD characteristics, and 13.3% (*n* = 847) presented good MD characteristics. The total FINDRISC, MSI and MSAF scores of the participants were 7.0 ± 4.66, 37.0 ± 13.02 and 6.0 ± 2.87, respectively, and the majority of them had low T2DM (62.6%), 49.8% had a moderate risk of MetS (according to the MSAF), and 21.3% (according to the MSI) had a risk of MetS (Table 2). As the level of MD characteristics increased, the SHEBS (3.0 ± 0.96 vs. 3.3 ± 0.94 vs. 3.6 ± 1.04) and SFLS (118.8 ± 27.08 vs. 122.2 ± 27.05 vs. 126.6 ± 28.12) scores also increased significantly (*p* < 0.001) (Table 2). Participants exhibiting high levels of MD traits had lower MSI scores than those exhibiting medium and low levels (35.3 ± 14.18 vs. 37.7 ± 12.63 and 37.0 ± 12.88, *p* < 0.001), whereas those exhibiting low levels of MD traits had significantly higher MSAF scores than those exhibiting medium and high levels (6.2 ± 2.77 vs. 5.9 ± 2.87 and 5.7 ± 3.03, *p* < 0.001). In terms of the MD features, no significant difference was found between the groups in terms of the FINDRISC score (Table 2).

**Table 1:** General characteristics of the participants.

General characteristics		Total (*n* = 6,364)	*p*
Gender *n* (%)	Female	3,847 (60.4)	<0.001
	Male	2,517 (39.6)	
Marital status *n* (%)	Married	1,673 (26.3)	<0.001
	Single	4,691 (73.7)	
Education *n* (%)	Primary school	584 (9.2)	<0.001
	High school	3,991 (62.7)	
	University	1,692 (26.6)	
	Postgraduate	97 (1.5)	
Income status *n* (%)	Under minimum wage	107 (8.5)	<0.001
	Minimum wage	3,543 (55.7)	
	Above the minimum wage	2,276 (35.8)	
Occupation *n* (%)	Laborer	319 (5.0)	<0.001
	Officer	562 (8.8)	
	Employee in the business	856 (13.5)	
	Independent business	286 (4.5)	
	Retired	247 (3.9)	
	Student	3,434 (54.0)	
	Unemployed	660 (10.4)	
Smoking *n* (%)	Yes	2,311 (36.3)	<0.001
	No	4,053 (63.7)	
Alcohol *n* (%)	Yes	1,039 (16.3)	<0.001
	No	5,325 (83.7)	
Physical activity status *n* (%)	Inactive	4,937 (77.6)	<0.001
	Active	1,427 (22.4)	
Age (years, Mean ± SD)		27.0 ± 10.24	
Body mass index (kg/m^2^, Mean ± SD)		24.2 ± 4.37	
Waist circumference (cm, Mean ± SD)		82.0 ± 13.24	
Physical activity score (Mean ± SD)		2.1 ± 2.12	

**Table 2:** Participants’ levels of sustainable healthy eating behaviors, sustainable food literacy levels, and diabetes and metabolic syndrome risk status.

Sustainable healthy eating behaviors, sustainable food literacy levels, and diabetes and metabolic syndrome risk			MEDAS			
		Total	Low	Moderate	High	*p* value
		(*n* = 6,364)	(*n* = 1,537)	(*n* = 3,980)	(*n* = 847)	
SHEBS (Mean ± SD)	Total score	3.3 ± 0.97	3.0 ± 0.96^a^	3.3 ± 0.94^b^	3.6 ± 1.04^c^	<0.001
	Quality labels	3.2 ± 1.11	2.9 ± 1.08^a^	3.2 ± 1.07^b^	3.6 ± 1.15^c^	<0.001
	Seasonal food & avoiding food waste	3.6 ± 1.09	3.3 ± 1.08^a^	3.7 ± 1.06^b^	3.9 ± 1.15^c^	<0.001
	Animal welfare	3.3 ± 1.33	2.9 ± 1.34^a^	3.3 ± 1.28^b^	3.5 ± 1.36^c^	<0.001
	Meat reduction	3.0 ± 1.28	2.7 ± 1.27^a^	3.1 ± 1.24^b^	3.4 ± 1.34^c^	<0.001
	Healthy and balanced nutrition	3.9 ± 1.30	3.6 ± 1.26^a^	3.9 ± 1.28^b^	4.2 ± 1.37^c^	<0.001
	Local food	3.0 ± 1.38	2.6 ± 1.37^a^	3.0 ± 1.34^b^	3.4 ± 1.43^c^	<0.001
	Low fat	4.0 ± 1.34	3.8 ± 1.36^a^	4.1 ± 1.30^b^	4.3 ± 1.41^c^	<0.001
SFLS (Mean ± SD)	Total score	122.0 ± 27.30	118.8 ± 27.08^a^	122.2 ± 27.05^b^	126.6 ± 28.12^c^	<0.001
	Sustainable food knowledge I	26.0 ± 7.88	25.2 ± 7.75^a^	26.0 ± 7.88^b^	27.1 ± 7.95^c^	<0.001
	Sustainable food knowledge II	20.5 ± 5.25	20.1 ± 5.33^a^	20.6 ± 5.15^b^	21.0 ± 5.53^b^	<0.001
	Food and culinary skills	30.6 ± 8.14	30.1 ± 8.18^a^	30.6 ± 8.12^a^	31.6 ± 8.09^b^	<0.001
	Attitudes	13.5 ± 4.41	13.2 ± 4.48^a^	13.6 ± 4.38^b^	14.0 ± 4.38^c^	<0.001
	Intention to take action and strategies to take action	31.1 ± 9.53	30.0 ± 9.72^a^	31.2 ± 9.41^b^	32.8 ± 9.52^c^	<0.001
FINDRISC total score (Mean ± SD)		7.0 ± 4.66	7.0 ± 4.41	7.0 ± 4.67	6.8 ± 5.06	0,35
MSI total score (Mean ± SD)		37.0 ± 13.02	37.7 ± 12.63^a^	37.0 ± 12.88^a^	35.3 ± 14.18^b^	<0.001
MSAF total score (Mean ± SD)		6.0 ± 2.87	6.2 ± 2.77^a^	5.9 ± 2.87^b^	5.7 ± 3.03^b^	<0.001
FINDRISC classification *n* (%)	Very low risk	3,981 (62.6)	964 (62.7)	2,493 (62.6)	524 (61.9)	0.29
	Low risk	1,365 (21.4)	348 (22.6)	834 (21.0)	183 (21.6)	
	Moderate risk	527 (8.3)	117 (7.6)	349 (8.8)	61 (7.2)	
	High risk	391 (6.1)	90 (5.9)	239 (6.0)	62 (7.3)	
	Very high risk	100 (1.6)	18 (1.2)	65 (1.6)	17 (2.0)	
MSI classification *n* (%)	No or low risk	5,007 (78.7)	1,195 (77.7)	3,131 (78.7)	681 (80.4)	0.31
	Risk	1,357 (21.3)	342 (22.3)	849 (21.3)	166 (19.6)	
MSAF classification *n* (%)	Low	1,942 (30.5)	396 (25.8)	1,238 (31.1)	308 (36.4)	<0.001
	Moderate	3,172 (49.8)	802 (52.2)	1,991 (50.0)	379 (44.7)	
	High	1,250 (19.6)	339 (22.1)	751 (18.9)	160 (18.9)	
Physical activity *n* (%)	Inactive	4,937 (77.6)	1,242 (80.8)	3,063 (77.0)	632 (74.6)	0.001
	Active	1,427 (22.4)	295 (19.2)	917 (23.0)	215 (25.4)	

χ^2^, Chi-square analysis was applied to categorical data. One-Way Analysis of Variance (One-Way ANOVA) was used to analyze numerical data. Lettering was done according to post hoc test results and shows significant differences between groups. FINDRISC, Finnish Diabetes Risk Scale; MEDAS, Mediterranean Diet Adherence Screener; MSAF, Metabolic Syndrome Research Form; MSI, Metabolic Syndrome Index; SFLS, Sustainable Food Literacy Scale; SHEBS, Sustainable and Healthy Eating Behaviors Scale.

**Figure 1:**
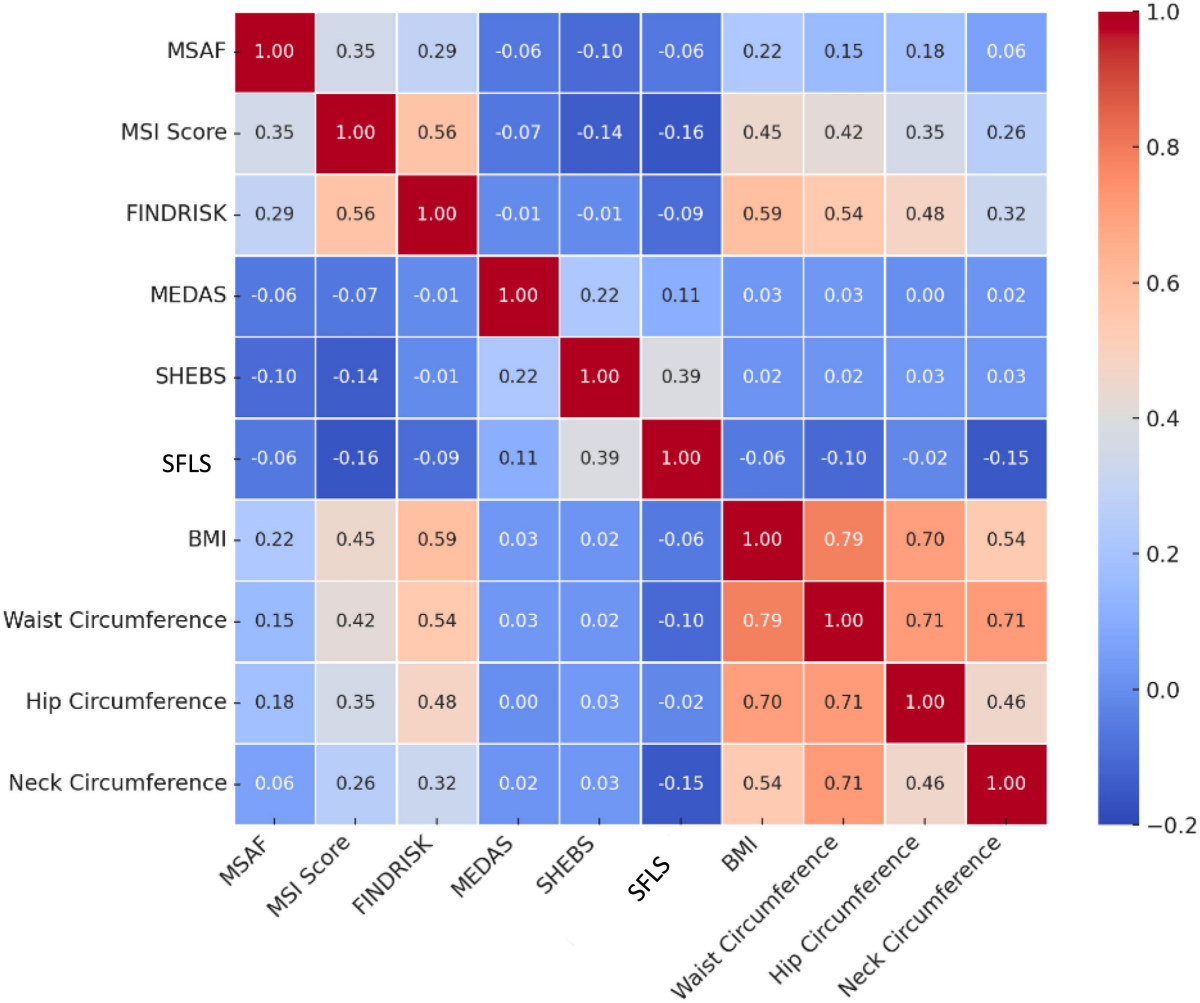
Correlation matrix. Correlation coefficients are based on Pearson correlation. FINDRISC, Finnish Diabetes Risk Scale; MEDAS, Mediterranean Diet Adherence Screener; MSAF, Metabolic Syndrome Research Form; MSI, Metabolic Syndrome Index; SFLS, Sustainable Food Literacy Scale; SHEBS, Sustainable and Healthy Eating Behaviors Scale.

The relationships between sustainable healthy eating behaviors, sustainable food literacy, and MD traits and the risk of T2DM and MetS are presented in Table 3 and Figure 1. MSI and MSAF were significantly negatively and weakly correlated with SHEBS (r = −0.01 and r = −0.10, respectively), SFLS (r = −0.16 and r = −0.06, respectively) and MEDAS (r = −0.06 and r = −0.06, respectively) (*p* < 0.001) and negatively and weakly correlated with only the FINDRISC score and SFLS (r = −0.09, *p* < 0.001) (Table 3). In addition to a positive significant relationship between SFLS and SHEBS (r = 0.38) and MEDAS (r = 0.11), a positive relationship was found between SHEBS and MEDAS (r = 0.22) (*p* < 0.001) (Table 3). As the participants’ MSI (SHEBS: 3.1 ± 1.05 to 3.3 ± 0.95; SFLS: 115.7 ± 27.44 to 123.7 ± 27.02), MSAF (SHEBS: 3.2 ± 0.99 to 3.4 ± 1.00; SFLS: 120.5 ± 27.18 to 124.8 ± 28.26) and FINDRISC (SHEBS: 3.2 ± 0.99 to 3.6 ± 1.15; SFLS: 117.3 ± 29.05 to 123.6 ± 26.99) risk levels decreased, the SHEBS and SFLS scores significantly increased (*p* < 0.001) (Table 4). As the MSAF risk level increased, the MEDAS score of the participants significantly decreased (7.3 ± 2.34 vs. 7.0 ± 2.32, 6.9 ± 2.38; *p* < 0.001) (Table 4).

**Table 3:** The relationship between sustainable healthy eating behaviors, sustainable food literacy, and Mediterranean diet characteristics and the risk of diabetes and metabolic syndrome.

Sustainable healthy eating behaviors, sustainable food literacy, and Mediterranean diet characteristics		FINDRISC total score		MSI total score		MSAF total score		SHEBS total score		SFLS total score		MEDAS total score	
		r	*p*	r	*p*	r	*p*	r	*p*	r	*p*	r	*p*
SHEBS	Total score	−0.008	0.52	−0.01	<0.001	−0.10	<0.001	1.000	-	0.38	<0.001	0.22	<0.001
	Quality labels	−0.01	0.28	−0.13	<0.001	−0.10	<0.001	0.77	<0.001	0.35	<0.001	0.21	<0.001
	Seasonal food & avoiding food waste	0.04	0.001	−0.07	<0.001	−0.05	<0.001	0.75	<0.001	0.35	<0.001	0.17	<0.001
	Animal welfare	−0.01	0.34	−0.10	<0.001	−0.09	<0.001	0.68	<0.001	0.26	<0.001	0.18	<0.001
	Meat reduction	0.01	0.17	−0.05	<0.001	−0.09	<0.001	0.59	<0.001	0.19	<0.001	0.18	<0.001
	Healthy and balanced nutrition	−0.05	<0.001	−0.17	<0.001	−0.10	<0.001	0.73	<0.001	0.40	<0.001	0.17	<0.001
	Local food	−0.01	0.16	−0.10	<0.001	−0.07	<0.001	0.62	<0.001	0.20	<0.001	0.19	<0.001
	Low fat	0.005	0.69	−0.09	<0.001	−0.05	<0.001	0.65	<0.001	0.33	<0.001	0.12	<0.001
SFLS	Total score	−0.09	<0.001	−0.16	<0.001	−0.06	<0.001	0.38	<0.001	1.000	-	0.11	<0.001
	Sustainable food knowledge I	−0.11	<0.001	−0.16	<0.001	−0.08	<0.001	0.29	<0.001	0.76	<0.001	0.09	<0.001
	Sustainable food knowledge II	−0.05	<0.001	−0.09	<0.001	−0.03	0.004	0.29	<0.001	0.77	<0.001	0.06	<0.001
	Food and culinary skills	−0.02	0.05	−0.06	<0.001	−0.01	0.29	0.29	<0.001	0.77	<0.001	0.07	<0.001
	Attitudes	−0.09	<0.001	−0.13	<0.001	−0.05	<0.001	0.25	<0.001	0.69	<0.001	0.07	<0.001
	Intention to take action and strategies to take action	−0.007	<0.001	−0.148	<0.001	−0.05	<0.001	0.33	<0.001	0.81	<0.001	0.10	<0.001
MEDAS total score		−0.009	0.46	−0.06	<0.001	−0.06	<0.001	0.22	<0.001	0.11	<0.001	1.000	-

r, Pearson correlation coefficient; FINDRISC, Finnish Diabetes Risk Scale; MEDAS, Mediterranean Diet Adherence Screener; MSAF, Metabolic Syndrome Research Form; MSI, Metabolic Syndrome Index; SFLS, Sustainable Food Literacy Scale; SHEBS, Sustainable and Healthy Eating Behaviors Scale.

**Table 4:** Sustainable healthy eating behaviors, sustainable food literacy, and Mediterranean diet characteristics according to participants’ metabolic syndrome and diabetes risk classifications.

sustainable healthy eating behaviors, sustainable food literacy, and Mediterranean diet characteristics		FINDRISC						MSI			MSAF			
		Very low risk (*n* = 3,981)	Low risk (*n* = 1,365)	Moderate risk (*n* = 527)	High risk (*n* = 391)	Very high risk (*n* = 100)	*p*-value	No or low risk (*n* = 5,007)	Risk (*n* = 1,357)	*p*-value	Low (*n* = 1,942)	Moderate (*n* = 3,172)	High (*n* = 1,250)	*p*-value
SHEBS	Total score	3.6 ± 1.15	3.4 ± 1.07	3.3 ± 0.97	3.3 ± 0.96	3.2 ± 0.99	<0.001	3.3 ± 0.95	3.1 ± 1.05	<0.001	3.4 ± 1.00^a^	3.2 ± 0.94^b^	3.2 ± 0.99^c^	<0.001
	Quality labels	3.5 ± 1.12	3.4 ± 1.26	3.1 ± 1.09	3.2 ± 1.09	3.1 ± 1.1	<0.001	3.2 ± 1.09	3.0 ± 1.16	<0.001	3.4 ± 1.12^a^	3.1 ± 1.08^b^	3.1 ± 1.11^b^	<0.001
	Seasonal food & avoiding food waste	4.1 ± 1.24	3.8 ± 1.12	3.6 ± 1.09	3.6 ± 1.08	3.6 ± 1.12	<0.001	3.6 ± 1.08	3.6 ± 1.14	0.04	3.7 ± 1.12^a^	3.6 ± 1.05^b^	3.6 ± 1.13^b^	<0.001
	Animal welfare	3.6 ± 1.32	3.4 ± 1.37	3.2 ± 1.33	3.3 ± 1.32	3.1 ± 1.33	0.006	3.3 ± 1.32	3.1 ± 1.35	<0.001	3.4 ± 1.31^a^	3.2 ± 1.31^b^	3.1 ± 1.35^b^	<0.001
	Meat reduction	3.3 ± 1.4	3.2 ± 1.38	3.1 ± 1.28	3.0 ± 1.26	3.0 ± 1.27	0.002	3.1 ± 1.27	3.0 ± 1.31	0.009	3.2 ± 1.26^a^	3.0 ± 1.28^b^	2.9 ± 1.28^c^	<0.001
	Healthy and balanced nutrition	4.2 ± 1.32	3.8 ± 1.41	3.8 ± 1.30	3.9 ± 1.28	3.7 ± 1.32	<0.001	4.0 ± 1.27	3.6 ± 1.36	<0.001	4.1 ± 1.33^a^	3.8 ± 1.27^b^	3.7 ± 1.29^b^	<0.001
	Local food	3.3 ± 1.59	3.1 ± 1.45	3.0 ± 1.41	3.0 ± 1.35	2.8 ± 1.38	0.001	3.0 ± 1.36	2.8 ± 1.44	<0.001	3.1 ± 1.36^a^	2.9 ± 1.37^b^	2.9 ± 1.40^b^	<0.001
	Low fat	4.4 ± 1.53	4.2 ± 1.45	4.0 ± 1.32	4.0 ± 1.32	4.0 ± 1.35	0.02	4.1 ± 1.32	3.9 ± 1.38	<0.001	4.2 ± 1.32^a^	4.0 ± 1.32^b^	4.0 ± 1.37^b^	<0.001
SFLS	Total score	123.6 ± 26.99	120.3 ± 26.82	119.1 ± 27.71	117.6 ± 28.3	117.3 ± 29.05	<0.001	123.7 ± 27.02	115.7 ± 27.44	<0.001	124.8 ± 28.26^a^	120.8 ± 26.62^b^	120.5 ± 27.18^b^	<0.001
	Sustainable food knowledge I	26.5 ± 7.78	25.7 ± 7.78	22.7 ± 8.27	24.5 ± 7.93	24.5 ± 8.40	<0.001	26.5 ± 7.76	24.2 ± 8.06	<0.001	27.0 ± 7.97^a^	25.6 ± 7.83^b^	25.4 ± 7.73^b^	<0.001
	Sustainable food knowledge II	20.7 ± 5.18	20.3 ± 5.29	20.3 ± 6.00	20.1 ± 5.34	20.1 ± 5.44	0,006	20.7 ± 5.19	19.8 ± 5.41	<0.001	20.9 ± 5.26^a^	20.4 ± 5.28^b^	20.3 ± 5.14^b^	0,001
	Food and culinary skills	30.8 ± 8.00	30.3 ± 8.14	32.9 ± 7.91	30.1 ± 8.64	30.0 ± 8.86	0,006	30.8 ± 8.07	29.8 ± 8.38	<0.001	30.9 ± 8.20	30.4 ± 8.09	30.7 ± 8.18	0,11
	Attitudes	13.8 ± 4.36	13.3 ± 4.34	13.0 ± 4.60	12.9 ± 4.51	12.5 ± 4.68	<0.001	13.8 ± 4.36	12.6 ± 4.48	<0.001	13.9 ± 4.43^a^	13.4 ± 4.38^b^	13.4 ± 4.41^b^	<0.001
	Intention to take action and strategies to take action	31.7 ± 9.37	30.5 ± 9.48	30.1 ± 11.22	29.9 ± 9.90	29.9 ± 10.02	<0.001	31.7 ± 9.35	29.0 ± 9.90	<0.001	31.9 ± 9.79^a^	30.9 ± 9.28^b^	30.5 ± 9.68^b^	<0.001
MEDAS total score		7.0 ± 2,36	7.0 ± 2.33	7.1 ± 2.24	7.2 ± 2.41	7.5 ± 2.24	0.09	7.0 ± 2.32	7.1 ± 2.35	0.15	7.3 ± 2.34^a^	7.0 ± 2.32^b^	6.9 ± 2.38^b^	<0.001

Independent Samples T-Test was used for variables with two groups, and the One-Way Analysis of Variance (One-Way ANOVA) test was used for variables with three groups or more. Lettering was based on post hoc test results and indicates significant differences between groups. FINDRISC, Finnish Diabetes Risk Scale; MEDAS, Mediterranean Diet Adherence Screener; MSAF, Metabolic Syndrome Research Form; MSI, Metabolic Syndrome Index; SFLS, Sustainable Food Literacy Scale; SHEBS, Sustainable and Healthy Eating Behaviors Scale.

**Figure 2:**
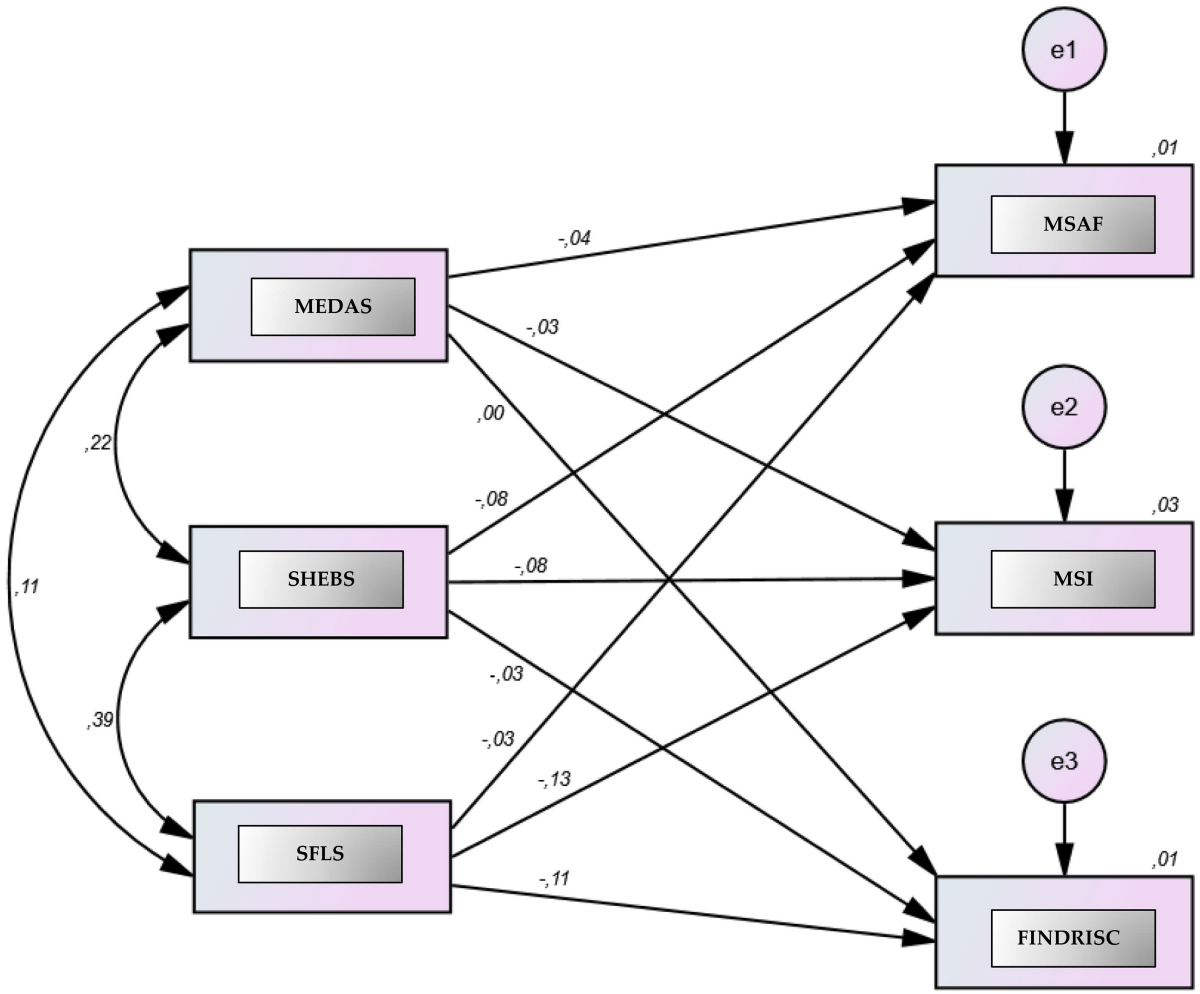
Path analysis examining the effects of MEDAS, SFLS, and SHEBS on MSI, MSAF, and FINDRISC. A path analysis based on structural equation modeling (SEM). The model tested whether three dependent variables were significantly predicted by MEDAS, SFLS, and SHEBS. The dependent variables were the MSI, MSAF, and FINDRISC scores, while the independent variables were the total scores of MEDAS, SFLS, and SHEBS. Two-way arrows represent the correlation (r) between the scales, and the one-way arrows represent the standardized beta coefficients (*β*). The term “e” represents the margin of error, whereas the number in the dependent variable represents the explanatory variance of the independent variables in the dependent variable.

The findings of the path analysis determining the effect level are presented in detail in Table 5, and a visual representation of the general structure of the model and the relationships between variables is given in Figure 2. SFLS and MEDAS had a negative and significant effect on the MSAF (*β* = −0.03, β = −0.04; *p* < 0.05, respectively), whereas SHEBS had a stronger negative and significant effect (*β* = −0.08; *p* < 0.001). These results indicate that exhibiting MD characteristics and sustainable healthy eating behaviors and being sustainable food literate reduce the risk level of MetS. When the MSI variable is examined, MEDAS (*β* = −0.03; *p* = 0.007), SHEBS (β = −0.08; *p* < 0.001) and especially SFLS (β = −0.13; *p* < 0.001) stand out as negative and significant predictors. These findings suggest that the risk of MetS decreases as adherence to a MD, sustainable healthy eating behaviors, and sustainable food literacy levels increase. In particular, the SFLS variable has the strongest effect on the MSI. While MEDAS had no significant effect on FINDRISC, SHEBS had a negative and significant effect (β = −0.03; *p* = 0.01). This suggests that as sustainable and healthy eating behaviors increased, the T2DM risk score also decreased slightly. SFLS was a significant negative predictor of FINDRISC scores (β = −0.11; *p* < 0.001).

**Table 5:** Path analysis examining the effects of MEDAS, SFLS, and SHEBS on MSI, MSAF, and FINDRISC.

Dependent variable	Path	Independent variable	B	S.E.	β (Beta)	*p*	R^2^
MSAF	←	MEDAS	−0.05	0.02	−0.04	0.001	0.012
MSAF	←	SHEBS	−0.24	0.04	−0.08	<0.001	
MSAF	←	SFLS	0.00	0.00	−0.03	0.04	
MSI	←	MEDAS	−0.19	0.07	−0.03	0.007	0.034
MSI	←	SHEBS	−1.12	0.18	−0.08	<0.001	
MSI	←	SFLS	−0.06	0.01	−0.13	<0.001	
FINDRISC	←	MEDAS	−0.01	0.03	−0.01	0.69	0.010
FINDRISC	←	SHEBS	0.16	0.07	−0.03	0.01	
FINDRISC	←	SFLS	−0.02	0.00	−0.11	<0.001	

B, Unstandardized path coefficient; β (Beta), Standardized path coefficient; R^2^, Proportion of variance explained in dependent factor. FINDRISC, Finnish Diabetes Risk Scale; MEDAS, Mediterranean Diet Adherence Screener; MSAF, Metabolic Syndrome Research Form; MSI, Metabolic Syndrome Index; SFLS, Sustainable Food Literacy Scale; SHEBS, Sustainable and Healthy Eating Behaviors Scale.

## 4 Discussion

It has been reported that MD may reduce the risk of MetS due to its content of complex carbohydrates, polyphenols such as resveratrol, naringenin, kaempferol, apigenin, hesperidin, oleuropein, ellagic acid, rosmarinic acid, quercetin, dietary fiber, polyunsaturated fatty acids such as omega 3 and 9, antioxidant vitamins (A, C, and E), and important minerals (calcium, potassium, magnesium, etc.) (29). This effect may be associated with a better lipid profile, lower blood pressure and blood glucose, and reduced rates of obesity (30). Similarly, anti-inflammatory and antioxidant compounds, glucagon-like peptide agonist compounds, and changes in the gut microbiota and MD traits may play important roles in the mechanisms related to T2DM (31). The current approach to MD focuses on examining MD traits in populations across geographic regions in relation to certain pathologies, including T2DM and MetS (30). In our study, participants with high levels of MD traits had significantly lower MSI and MSAF scores than did those with moderate and low levels, and MEDAS scores significantly decreased as MSAF risk levels increased. These findings confirm that individuals exhibiting MD traits have a lower risk of MetS. Path analysis revealed that MEDAS had a low negative effect on the MSAF (*β* = −0.04; *p* = 0.001) and MSI (β = −0.03; *p* = 0.007). It is possible that MEDAS is a significant negative predictor of MetS risk. These findings suggest that the risk of MetS decreases as adherence to a MD increases. Conversely, our study revealed that MEDAS was not a significant predictor of T2DM risk. MD characteristics and adherence to healthy eating guidelines were found to be associated with a reduced likelihood of developing MetS in the Luxembourgish population (n = 1,404) (32). A study evaluating 2007–2020 NHANES data from the American population (20,991) reported a negative association between the MD score and MetS prevalence (33). In another study examining 3,417 participants (mean age: 60.1 ± 9.14), according to the results of multiple regression analysis, the MD score was negatively related to the number of MetS components (*β* = −0.336), and the higher the level of MD features, the lower the probability of developing MetS (30). In the aforementioned study, the effect of MD was found to be greater than that in our study. This effect is possibly due to the sample differences between studies. Our study sample consisted of a younger population (mean age 27 years), which may have influenced the potential effect of MD due to the lower risk of MetS (risk according to MSI: 21.3%). Furthermore, the majority of participants exhibited moderate MD features, and the proportion with good MD features was quite low. This may have influenced the results. While no significant MEDAS effect was observed on T2DM risk in our study, other studies have reported a strong inverse linear relationship between MD and T2DM (34–36). In the cohort study, the incidence of diabetes was 2.9 versus 4.8 per 100 person-years in participants with high and low/moderate adherence to the Mediterranean diet (37). Similarly, in a dose–response meta-analysis of prospective cohort studies, each 1-point increase in Mediterranean diet score was associated with a 3% reduced risk of diabetes (HR = 0.97; *p* < 0.001) (38). This difference may be due to differences in study methodology, sample selection, and study type. Geographic region, country of residence, genetics, and lifestyle behavior and culture may be determinants of MetS and T2DM risk. Ethnic differences across studies should not be overlooked when interpreting them. Notably, this study was conducted among young adults in Türkiye, and cultural, nutritional, and lifestyle characteristics may influence health outcomes. The fact that the sample of our study was young and had low risk of diabetes, the moderate level of Mediterranean diet characteristics (62.5%), and the underrepresentation of high and low levels may have influenced these results. It may be beneficial to conduct the study in a sample where the diabetes risk distribution is homogeneous and where various age groups are represented. To reach definitive conclusions, it is crucial to conduct large-sample, comprehensive studies across geographic regions and countries, examining various age groups in detail. Furthermore, it is crucial that the management of MetS and T2DM incorporates diet, exercise, and lifestyle changes. In conclusion, MD features may have a positive effect on MetS parameters, but this effect may vary from one component to another and may differ across studies because of the included volunteers, the analyses performed, methodological differences, heterogeneity and associated pathologies. The lack of research on this issue in the Turkish population is a shortcoming, and further research is needed in this area. For these reasons, the main novelty of this study is that, to our knowledge, it is the first study to holistically analyze the impact of MD traits on the risk of MetS and T2DM in the Turkish population, incorporating sustainability concepts (sustainable food literacy and sustainable healthy eating behaviors) via validated scales.

Sustainable nutrition, sustainable consumption behaviors, and food literacy should be considered as a whole (39). Higher digital health nutrition literacy was associated with an increase in environmentally responsible food choices (*β* = 0.28, *p* < 0.001) and an approximately 1.04-fold increase in the likelihood of exhibiting MD traits (*p* < 0.001) (40). Since nutrition literacy is the ability to critically evaluate food-related information and act on this information (41), increasing the level of nutritional literacy and promoting sustainable dietary behaviors can be effective strategies for managing and reducing the prevalence of chronic diseases (8). In a recent study (*n* = 3,146), nutritional literacy and sustainable healthy eating behaviors were found to be significantly lower in a Turkish population with higher CVD risk and FINDRISC scores (*p* < 0.05) (8). In another study, dietary behavior patterns, perceived food literacy, and fasting blood glucose levels explained 33.2% of the variance in glycated hemoglobin A1c levels in a Turkish population with T2DM (*n* = 240). It was concluded that food literacy and dietary behaviors should be improved in the Turkish population with T2DM to improve glycemic control and lipid profiles (42). In our study, SFLS had a significant and strong negative effect on both MSI and FINDRISC and a significant and strong positive effect on SHEBS and MEDAS, indicating that increasing the sustainable food literacy level reduced the risk of MetS and T2DM by supporting SHEBS and MEDAS. Sustainable dietary patterns and eating behaviors can have beneficial effects on human health as well as positive impacts on the environment. Promoting a healthier diet by supporting the consumption of environmentally sensitive, culturally appropriate, and nutritious local and seasonal foods is believed to be an important step in preventing chronic diseases (9, 10). Food literacy has been identified as a predictor of achieving optimal health and sustainability (43). Considering the impact of health and food literacy on sustainable diets (SDs) and food choices, the SFLS may reduce the risk of MetS and T2DM by supporting sustainable dietary patterns and nutritional behaviors. Because there are no studies examining these issues in detail in the Turkish population or globally, it is impossible to reach a definitive conclusion. Longitudinal studies with large sample sizes and randomized controlled trials are needed to clarify potential causal relationships. Furthermore, addressing food literacy in multidisciplinary T2DM education and management programs can improve important health outcomes (44). To improve glycemic control and lipid profiles in individuals with T2DM, it is recommended not only to increase food literacy levels but also to implement interventions that support the translation of this knowledge into healthy eating behaviors and the long-term sustainability of these behaviors (42). These risks can be minimized by ensuring that sustainable food literacy is widespread throughout society. Sustainable food literacy can encourage individuals to adopt healthy lifestyles and sustainable healthy eating behaviors and to develop them throughout their lives.

In the Diabetes Prevention Program, 38% of participants who met the criteria for MetS through lifestyle modification were shown to no longer exhibit features of the syndrome over a mean follow-up of 3.2 years (45). It has been reported that long-term participation in lifestyle modifications in the early stages of MetS can reverse MetS (45). Similarly, T2DM can be prevented through lifestyle changes (45). In particular, healthy eating habits, which are healthy lifestyle behaviors, have been shown to reduce the risk of developing CVD, T2DM, and MetS (46, 47). According to the results of a meta-analysis conducted in the French population (16,358 participants from the NutriNet-Santé), a higher consumption of healthy plant foods was associated with a lower likelihood of developing MetS (men: 0.85; women: 0.72, 95% CI) (48). Another dietary approach that stands out among sustainability concepts is the planetary diet, and it is suggested that this approach may have an important role in reducing the risk of T2DM, improving glycemic control, and may be an effective strategy for managing and preventing MetS and CVD (49). Similarly, a five-year follow-up study (*n* = 891) revealed that greater adherence to a planetary healthy diet was associated with lower cardiometabolic risk factors and the risk of developing T2DM in South Asians living in the Americas (50). Supporting evidence suggests that greater adherence to a diet designed to support environmental and human health and the EAT-Lancet dietary pattern may help prevent the incidence of T2DM (51–53). In our study, the SHEBS was found to be a negative and significant predictor of MetS risk (*β* = −0.08 for MSAF and MSI; *p* < 0.001). Its effect on the risk of T2DM is low, with a significant negative effect. The lower effect of the SHEBS may be attributed to the younger age of our study population (mean age 27) and the lower risk of MetS and T2DM. Because this issue is current and new, few studies have investigated the relationships between sustainable healthy eating behaviors and these risks. Current studies address different sustainable healthy dietary approaches. The inadequacy of the studies and the existing methodological and sampling differences between studies limit the interpretation of the relevant data and the ability to make clear judgments. However, the data from our study confirm that sustainable healthy eating behaviors may have beneficial effects on the risk of MetS and T2DM. As a result, interventions should be implemented to support the adoption of healthy eating behaviors and the long-term sustainability of these behaviors.

This study has several limitations and strengths. The primary limitation is the analysis of cross-sectional data, which limits our ability to establish causality. This cross-sectional study cannot establish causal relationships, limiting inference of directionality. Because the study was conducted in a specific population in Türkiye, the generalizability of the findings may be limited. Since participation in our study was voluntary, the sample was intended to be representative of the adult population in Türkiye; however, it was acknowledged that there may be limitations in representation due to the sampling method. The young population and high student ratio in our study may limit external validity, and it would be beneficial to conduct such studies in older and more diverse populations. The sample of our study consists of a young population (average: 27 years old) and the results obtained cannot be generalized to the entire Turkish society. Longitudinal studies and randomized controlled trials are needed to clarify potential causal relationships. Furthermore, the risk of MetS and T2DM has been estimated via validated risk scores rather than clinical diagnoses. While these scores are widely used, they may not fully reflect the true disease incidence. Another potential limitation is that the data were based on participant self-reports. Data based on self-declaration may not reflect the normal situation or may result in misrepresentation. Obtaining anthropometric measurements (body weight, height, BMI, and waist circumference) through self-report may underestimate the prevalence of obesity and the distribution of metabolic risks. We emphasize that anthropometric measurements should be taken by research experts and recommend that this issue be taken into consideration in future studies. The strengths of the study include the sample size and strict adherence to standardized conditions for all criteria defining MetS and T2DM risk. To our knowledge, no study has investigated this issue in the Turkish population, and its unique nature stands out as another strength. This study is the first to holistically analyze the impact of MD traits on the risk of MetS and T2DM in the Turkish population, incorporating sustainability concepts, which is another strength of our study.

## 5 Conclusion

The data from this study confirm the role of sustainable healthy eating behaviors, sustainable food literacy, and MD in preventing MetS and highlight that sustainable food literacy is the most significant negative predictor of T2DM risk. It is crucial to consider sustainable healthy eating behaviors, sustainable food literacy, and sustainable diets, such as MD, as a whole in MetS and T2DM prevention strategies. The importance of sustainable healthy eating patterns should be emphasized, and public awareness activities should be conducted. In this context, activities aimed at increasing sustainable food literacy should be undertaken throughout the population. In addition, there is a need to conduct such studies in Turkish society to investigate and illuminate this issue in depth.

## Data Availability

The datasets generated and/or analyzed during the current study are not publicly available owing to restrictions (e.g., they contain information that could compromise the privacy of research participants) but are available from the corresponding author upon reasonable request. Requests to access the datasets should be directed to HB, handecekici@hotmail.com.
